# Elevated Plasma microRNA-105-5p Level in Patients With Idiopathic Parkinson’s Disease: A Potential Disease Biomarker

**DOI:** 10.3389/fnins.2019.00218

**Published:** 2019-03-18

**Authors:** Zhaofei Yang, Tianbai Li, Yanhua Cui, Song Li, Cheng Cheng, Bairong Shen, Weidong Le

**Affiliations:** ^1^Center for Clinical Research on Neurological Diseases, The First Affiliated Hospital, Dalian Medical University, Dalian, China; ^2^Liaoning Provincial Key Laboratory for Research on the Pathogenic Mechanisms of Neurological Diseases, The First Affiliated Hospital, Dalian Medical University, Dalian, China; ^3^International Education College, Dalian Medical University, Dalian, China; ^4^Institute for Systems Genetics, West China Hospital, Sichuan University, Chengdu, China

**Keywords:** bioinformatics model, network vulnerability analysis, idiopathic Parkinson’s disease, microRNA-105-5p, biomarker

## Abstract

Parkinson’s disease (PD) is the second most common neurodegenerative disease, which still lacks a biomarker to aid in diagnosis and to differentiate diagnosis at the early stage of the disease. microRNAs (miRNAs) are small and evolutionary conserved non-coding RNAs that are involved in post-transcriptional gene regulation. Several miRNAs have been proposed as potential biomarkers in several diseases. In the present study, we screened miRNAs using a network vulnerability analysis, to evaluate their potential as PD biomarkers. We first extracted miRNAs that were differentially expressed between PD and healthy controls (HC) samples. Then we constructed the PD-specific miRNA-mRNA network and screened miRNA biomarkers using a new bioinformatics model. With this model, we identified miR-105-5p as a putative biomarker for PD. Moreover, we measured miR-105-5p levels in the plasma of patients with idiopathic PD (IPD) (*n* = 319), neurological disease controls (NDC, *n* = 305) and HC (*n* = 273) using reverse transcription real-time quantitative PCR (RT-qPCR). Our data clearly demonstrated that the plasma miR-105-5p level in IPD patients was significantly higher than those of HC (251%, *p* < 0.001) and NDC (347%, *p* < 0.001). There was no significant difference in miR-105-5p expression between IPD patients with or without anti-PD medications. Interestingly, we found that the plasma miR-105-5p expression level may be able to differentiate IPD from parkinsonian syndrome, essential tremor and other neurodegenerative diseases. We believe that a change in the plasma miR-105-5p level is a potential biomarker for IPD.

## Introduction

Parkinson’s disease (PD) is the second most common neurodegenerative disease in the world (Sarkar et al., 2016; Xie and Chen, 2016), and the prevalence is on the rise, along with changing population demographics, which undoubtedly brings a heavy burden to both individual families and society (Sveinbjornsdottir, 2016). Although great achievements have been made to clarify the disease pathogenesis and to explore potent therapeutic strategies against PD for over 200 years, there is still no systematic understanding of this disease and current treatments are mostly symptomatic and lack the ability to prevent the disease occurrence or even to reverse the disease progression (Li and Le, 2017). In addition, the diagnosis of PD is mostly dependent on a clinical observation, and there is still a lack of reliable biomarkers to help make a correct diagnosis of the disease.

The complex process of molecular pathogenesis in PD also indicates that the disease likely results from multiple genetic and environmental factors during disease development and progression. Tremendous efforts have been made in the past to identify the neuropathological, biochemical, and genetic biomarkers of the disease so that a diagnosis could be established at earlier stages (Rachakonda et al., 2004). For years, a combination of microarrays and bioinformatics analytical tools have widely been used to identify differentially expressed genes and microRNAs (miRNAs) in PD and to identify differential diagnostic and prognostic markers (Chi et al., 2018; George et al., 2018; Su et al., 2018; Tan et al., 2018). MiRNAs are a class of ∼22 nt endogenous RNA molecules (Bartel, 2004; Yue et al., 2008). Mature miRNAs recognize and bind to the 3^′^ untranslated region of the messenger RNA (mRNA) by a specificity complementary sequence, which then affects the expression of target genes (Dong et al., 2016). MiRNAs have been reported to be encapsulated in microvehicles, which are secreted by circulating blood cells and other cells from different tissues (Zhang et al., 2010; Ding et al., 2016). Moreover, miRNAs have been proposed as putative non-invasive biomarkers in diagnosis, prognosis and response to treatment for several diseases, including neurodegenerative disorders (Batistela et al., 2017).

Research on human peripheral blood is common in clinical studies, in order to identify disease biomarkers and evaluate disease progression of PD and other neurodegenerative diseases. For instance, Martins et al. (2011) has reported that the expression levels of miR-199, miR-126, miR-151-5p, miR-29b/c, miR-335, miR-374a/b, miR-30b/c, miR-301a, miR-147, miR-28-5p, and miR-26a in peripheral blood mononuclear cells (PBMCs) from PD patients are significantly lower than those from healthy controls (HC) using microarrays. Plasma-based circulating miRNAs including miR-1826, miR450b-3p, miR-626, and miR-505 are differentially expressed in PD patients and controls and may serve as potential biomarkers for PD diagnosis (Khoo et al., 2012). Moreover, positive correlations between miR-221 and UPDRS-III, as well as between miR-221 and UPDRS-V exist in PD patients, suggesting that miRNAs can be used as a potential biomarker for detecting the stages of PD (Ma et al., 2016). Dong et al. (2015) proposed a panel of miR-143, miR-146a, miR-31, miR-93 as blood serum biomarkers for Alzheimer’s disease (AD). In addition, miR-338-3p, miR-451, miR-1275, miR-328, miR-638, miR-149, miR-665, miR-583 have been reported to be significantly changed in amyotrophic lateral sclerosis patients as compared to HC (De Felice et al., 2012). And plasma miR-34b is elevated in pre-manifest Huntington’s disease (Gaughwin et al., 2011). All these findings suggest that these miRNAs changes may be associated with PD and other neurodegenerative diseases.

Bioinformatics and biological tools are the main and efficient methods currently used in studies on miRNA biomarkers (Wang et al., 2017). However, it is still urgent to validate discovered potential miRNA biomarkers, based on bioinformatics analysis in a larger sample size, and to screen novel miRNA biomarkers. In the present study, we screened a potential PD biomarker, which has not been reported in this disease, using a bioinformatics method and identified miR-105-5p as a putative marker. We then recruited 319 patients with clinically diagnosed IPD, 273 HC and 305 patients with various non-PD neurological disorder controls (NDC). Plasma samples were collected to determine and validate the miR-105-5p expression level in IPD, HC, and NDC. The main aim of the present study is to determine whether the plasma miR-105-5p expression level is significantly altered in patients with IPD as compared with two groups of controls, and to evaluate whether age, gender, medications, and disease course affect the expression level of miR-105-5p.

## Materials and Methods

### Data Collection

The miRNA and mRNA expression dataset for biomarker prediction GSE16658 (Martins et al., 2011) and GSE6613 (Scherzer et al., 2007) were downloaded from gene expression omnibus (GEO) (Edgar et al., 2002). Among them, GSE16658 was generated by the miRCURY LNA microRNA Array, v.10.0 -hsa, mmu & rno, and contained miRNA expression data from PBMCs of 19 PD patients and 13 HC controls. The mRNA expression dataset GSE6613 was performed on the Afymetrix Human Genome U133A Array, including mRNA expression data from whole blood of 50 patients with PD and 23HC. The details are summarized in Table 1.

**Table 1:** Summary of the miRNA and mRNA dataset used in this study.

RNA type	GEO accession	Platform	Sample source	Number of samples (PD/controls)
miRNA	GSE16658	GPL7722	PBMCs	32(19/13)
mRNA	GSE6613	GPL96	Whole blood	73(50/23)


*GEO, gene expression omnibus; PBMCs, peripheral blood mononuclear cells.*

### Differentially Expressed miRNAs (DE-miRNAs) and mRNAs (DE-mRNAs) Extraction

The DE-miRNAs and DE-mRNAs were extracted based on the comparison of their expressions between PD and HC samples using a *t*-test. The Benjamini–Hochberg false discovery rate method was applied to adjust raw *p-*values (Benjamini and Hochberg, 1995). For the gene that is related to multiple probes, we designed it to the probe that had the most significant difference across its expression profile. The adjusted *p-*values (adj.*p-*value) < 0.05 and |log2 fold change| > 1 were chosen as the cut-off.

### PD-Specific miRNA-mRNA Network Construction

The PD-specific miRNA-mRNA network was constructed in two steps: First, a human miRNA-mRNA network (termed as the reference network) was built based on both experimentally validated and computationally predicted miRNA-mRNA regulatory data. Here the experimental data were mined from miRTarBase (version 4.5) (Hsu et al., 2014), TarBase (version 6.0) (Vergoulis et al., 2011), miRecords (version 4.0) (Xiao et al., 2008), and miR2Disease (Jiang et al., 2008) whereas the predicted data included information from HOCTAR (version 2.0) (Gennarino et al., 2011), ExprTargetDB (Gamazon et al., 2010), and starBase (version 2.0) (Li et al., 2013). To reduce the false positive rate, miRNA-mRNA pairs, validated by low-throughput experiments, e.g., real-time PCR, etc., were considered in this study, while the predicted pairs were selected only when they existed at least in two of the three computational prediction databases. In the second step, the DE-miRNAs and DE-mRNAs were mapped onto the reference, to extract the PD-specific miRNA-mRNA network.

### Refinement of DE-miRNA Lists With the Pipeline of Outlier miRNA Analysis

We used an in-house prediction model miRNA-BD (miRNA biomarker discovery) to remove false positive discoveries from the outlier miRNAs detected by the *t*-test. MiRNA-BD is a model created by Bairong Shen and his colleagues (Chen et al., 2013; Lin et al., 2018) to evaluate the relevance of miRNAs to given disease conditions. MiRNAs with unique regulatory activity will be screened as putative miRNA biomarkers for further analysis.

### Performance Evaluation

We performed the receiver-operating characteristic (ROC) analysis on both prediction and validation miRNA datasets, to evaluate the performance of identified miRNA biomarkers to classify PD and HC. The ROC curve and the area under curve (AUC) were drawn and calculated for each of the identified miRNAs using the R package “epicalc” (Chongsuvivatwong, 2012. Epicalc: epidemiological calculator). The percentage of the reported PD miRNA biomarkers in the predicted set was defined as the prediction precision to quantify the performance of the model.

### Participants and Blood Sampling

In this study, we recruited a total of 897 participants including 319 patients with IPD, 273 HC, and 305 from various non-PD NDC which consisted of 69 with epilepsy, 57 with cerebrovascular diseases, 49 with AD, 47 with parkinsonian syndromes including progressive supranuclear palsy, multiple system atrophy, dementia with Lewy bodies, corticobasal ganglionic degeneration and vascular parkinsonism, 22 with essential tremor (ET), 14 with myasthenia gravis, 14 with motor neuron disease, 11 peripheral neuropathy, seven with dementia, six with restless legs syndrome, three with migraine, three with multiple sclerosis, two with myelopathy and one with chorea minor. Detailed grouping information and sample sizes are summarized in Tables 2, 3. IPD patients were examined and diagnosed by at least two experienced neurologists from the First Affiliated Hospital of Dalian Medical University, according to the Movement Disorder Society Clinical Diagnostic Criteria for Parkinson’s Disease (Postuma et al., 2015). IPD disease severity was assessed by Modified Hoehn and Yahr (H-Y) staging. HC group were recruited from the Health Examination Center of the First Affiliated Hospital of Dalian Medical University, and the participants had no obvious neurological disorders or systemic diseases. All subjects (or their caregivers) recruited to our studies provided written informed consent, agreeing to participate in the project. This study has been granted ethical approval by the Ethics Committee of the First Affiliated Hospital of Dalian Medical University (approval number: LCKY2014-29).

**Table 2:** Demographic characteristics of subjects enrolled in the present study.

Groups	Number(%)	Gender male: female	*P*-value	*P*-value	Age (years) (mean ± SEM)	*P*-value	*P*-value
HC	273(30.4)	147:126	Ref		68.3 ± 9.6	Ref	
IPD	319(35.6)	176:143	NS	Ref	67.6 ± 9.6	NS	Ref
NDC	305(34.0)	166:139	NS	NS	63.6 ± 14.7	NS	NS


*Chi-square test and Student’s *t*-test. Ref, Reference; NS, not significant; HC, healthy controls; IPD, idiopathic Parkinson’s disease; NDC, neurological disease control.*

**Table 3:** Plasma miR-105-5p expression vs. internal control in all groups.

Groups	Number	miR-105-5p (mean ± SEM)	*P*-value	*P*-value
HC	273	0.065 ± 0.011	Ref	
IPD	319	0.163 ± 0.018	*P* < 0.001	Ref
NDC	305	0.047 ± 0.007	NS	*P* < 0.001
epilepsy	69	0.031 ± 0.009	NS	*P* < 0.001
cerebrovascular disease	57	0.028 ± 0.008	NS	*P* < 0.001
AD	49	0.035 ± 0.013	NS	*P* < 0.001
Parkinsonian syndrome	47	0.053 ± 0.012	NS	*P* < 0.001
ET	22	0.069 ± 0.051	NS	*P* < 0.05
myasthenia gravis	14	0.020 ± 0.014	NS	*P* < 0.001
motor neuron disease	14	0.031 ± 0.011	NS	*P* < 0.001
peripheral neuropathy	11	0.074 ± 0.039	NS	NS


*Ref, Reference; NS, not significant; HC, healthy controls; IPD, idiopathic Parkinson’s disease; NDC, neurological disease control; AD, Alzheimer’s disease; ET, essential tremor. This list displays the disease that numbers in group ≥10. Wilcoxon Rank-Sum (Mann–Whiney Test).*

Blood samples used in this study were collected by direct venipuncture at the First Affiliated Hospital of Dalian Medical University. Briefly, two milliliters of peripheral blood was drawn from the cubital vein into a vacuum blood tube with ethylenediaminetetraacetic acid (EDTA) and then the plasma was aliquoted (200 μL) into a sterile tube and stored at -80°C.

### Plasma miRNA Extraction and Quantification

Total miRNAs were extracted from plasma using a miRNA isolation system [Tiangen Biotech (Beijing) Co., Ltd., Beijing, China]. Five microliters of miRNA from plasma was reverse transcribed into first strand cDNA by a Tiangen miRCute miRNA cDNA synthesis kit [Tiangen Biotech (Beijing) Co., Ltd.]. The plasma miR-105-5p was determined by fluorescent real-time quantitative PCR (RT-qPCR). miR-16 was used as an internal control. Besides, the forward primer of miR-105-5p was commercialized and the reverse primer of miR-105-5p and miR-16 was provided by the miRcute miRNA qPCR Detection Kit [Tiangen Biotech (Beijing) Co., Ltd., Beijing, China]. The forward-primer targeting human miR-16 was as follows: 5^′^- ACA GAG AAG ATT AGC ATG GCC CCT G-3^′^. PCR was carried out using the ABI 7500 fast real-time PCR system (Applied Biosystems, Foster City, CA, United States) in a total volume of 20 μL for each reaction. Fluorescent reading from the qRT-PCR reaction was quantitatively analyzed by determining the difference of Ct (delta Ct) between Ct of miR-105-5p and internal control, and the miR-105-5p expression was determined by the formation of 2^-ΔCt^.

### Statistical Analysis

Quantitative data were expressed as mean ± SEM, or median depending on the distribution of the data. The chi-square test was used to evaluate the statistical differences of gender distribution between IPD patients and control subjects. The Student’s *t*-test or Mann–Whitney *U*-test was used to analyze differences between two groups in the age distribution and plasma miRNA level. A non-parametric ANOVA was performed using the GraphPad Prism software version 4 (GraphPad Inc., San Diego, CA, United States) to evaluate the differences in the mean value of the relative miRNA expression in individuals from each group. Exact 95% confidence intervals (CIs) were reported for the estimation of sensitivity and specificity. ROC curves were performed using MedCalc software version 17.2 (MedCalc Software Inc., Mariakerke, Belgium), and predictive performance of the putative biomarker (miRNA-105-5p) for the presence of IPD was quantified using AUC. Correlations were assessed using Spearman’s correlation coefficient (R). The correlations were reported at á level of 0.05. The other statistical analysis in this research was performed with the SPSS software version 13.0 (SPSS Inc., Chicago, IL, United States). All statistical checks were carried out two-sided and a *p*-value < 0.05 was considered as a statistical significance.

## Results

### Biomarker miRNAs for Predicting PD

**FIGURE 1:**
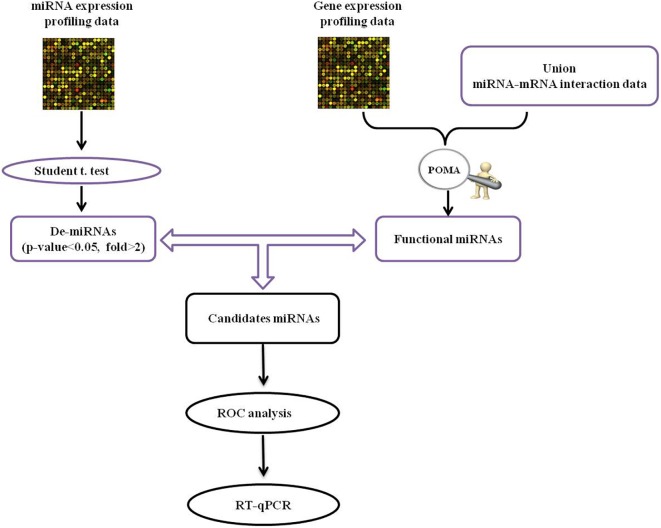
The schematic pipeline for PD miRNA biomarker identification. POMA, pipeline of outlier miRNA analysis; DE, differentially expressed; ROC, receiver operating characteristic curve; qRT-PCR, reverse transcription real-time quantitative PCR.

The human miRNA-mRNA network included 32739 regulatory pairs among 641 miRNAs and 7706 target genes/mRNAs. Based on the selected sample data, 20 DE-miRNAs and 976 DE-mRNAs were statistically identified. The schematic pipeline for PD miRNA biomarker identification is shown in Figure 1. According to the network vulnerability analysis, we found that miR-105-5p tends to have a high possibility and specificity in a PD-specific miRNA-mRNA network, suggesting that miR-105-5p may be a putative biomarker for predicting PD.

### Characteristics of Study Population

All 897 subjects enrolled in this study were Han ethnic Chinese with a male/female ratio of 489/408, respectively. The demographic characteristics of IPD patients and control subjects were summarized in Table 2. No significant difference in both gender and age was found among IPD, HC, and NDC groups.

### Plasma miR-105-5p Expression in All Study Groups

**FIGURE 2:**
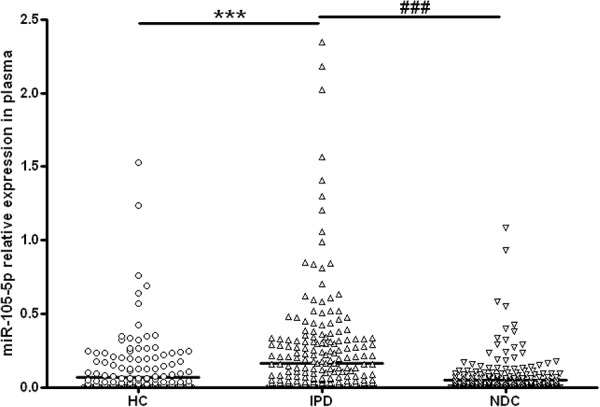
Scatter plots of miR-105-5p relative expression in plasma in different study groups. The level of miR-105-5p expression was significantly increased in patients with IPD (*n* = 319) as compared with healthy controls (HC, *n* = 273) and neurological disease controls (NDC, *n* = 305). Horizontal bars represent median value. Non-parametric ANOVA test, ^∗∗∗^*P*< 0.001 vs. HC, ^###^*P*< 0.001 vs. NDC.

As shown in Figure 2 and Table 3, the plasma miR-105-5p level in the IPD group was significantly higher than that in the HC group (0.163 ± 0.018 vs. 0.065 ± 0.011, *P* < 0.001) and the NDC group (0.163 ± 0.018 vs. 0.047 ± 0.007, *p* < 0.001). Interestingly, compared with the IPD group, our data also revealed a significant lower miR-105-5p level in the subgroups of NDC, including epilepsy, cerebrovascular disease, AD, Parkinsonian syndrome, ET, myasthenia gravis, motor neuron disease (Table 3).

### The Effects of Age and Gender on Plasma miR-105-5p Expression Level in IPD

**FIGURE 3:**
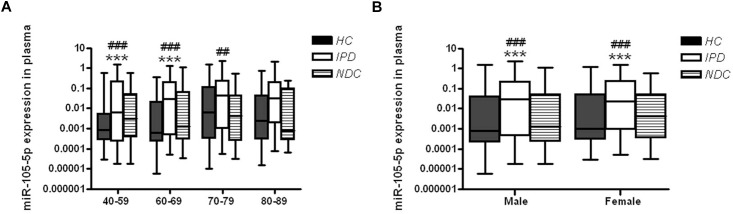
Plasma miR-105-5p expression levels in IPD patients and controls concerning the **(A)** age and **(B)** gender effects. Whiskers in the box plots represent minimum to maximum value. The center line in the box represents the median value. Non-parametric ANOVA test. ^∗∗∗^*P*< 0.001 vs. HC, ^##^*P*< 0.01 vs. NDC, ^###^*P*< 0.001 vs. NDC.

In order to explore the possible impacts of age on the changes of plasma miR-105-5p expression, IPD patients or controls were grouped into four subgroups, including 40∼59, 60∼69, 70∼79, and over 80 years. The plasma miR-105-5p levels of all these subgroups were determined and compared between IPD patients with controls. Specifically, two subgroups of IPD patients (40∼59 and 60∼69 years) showed significantly higher levels of plasma miR-105-5p than their age-matched control subjects (Figure 3A). In the subgroups over the age of 70 years old, the difference between the IPDs and HCs are not obvious. Much more interestingly, we also found differences in the plasma miR-105-5p level as shown by a dramatically higher level in both male and female, compared to same gender of HC and NDC (Figure 3B).

### The Impacts of Disease Duration and Severity on miR-105-5p Expression in IPD

**FIGURE 4:**
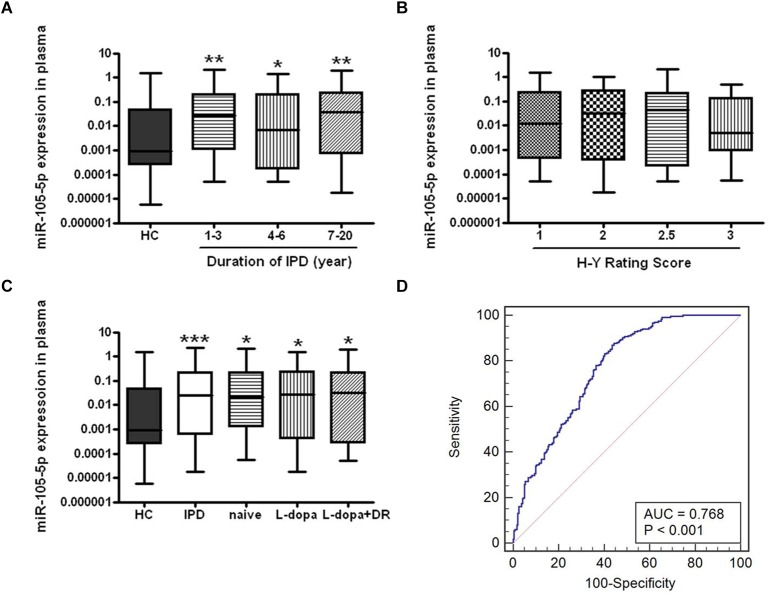
The expression level of miR-105-5p in IPD and HC. The effects of **(A)** disease course, **(B)** severity, and **(C)** anti-PD medications to the expression level of miR-105-5p. **(D)** ROC curve for miR-105-5p between PD and HC. The areas under the ROC curve (AUC) were 0.768 (95% CI, 0.727–0.805; *P* < 0.001). Whiskers in the box plots represent minimum to maximum value. The center line in the box represents the median value. Non-parametric ANOVA test, ^∗^*P*< 0.05 vs. HC, ^∗∗^*P*< 0.01 vs. HC, ^∗∗∗^*P*< 0.001 vs. HC.

We further analyzed disease duration and severity (H-Y score) in 319 patients with IPD. For disease duration, according to the years after onset of disease symptoms, IPD patients were classified into three subgroups (1∼3, 4∼6, and 7∼20 years). We found that plasma miR-105-5p expressions were significantly increased in these subgroups compared to the HC group, but were not obviously changed among the subgroups (Figure 4A). However, a similar association was not found between the miR-105-5p expression and H-Y scores (Figure 4B).

### The Influence of Medications on Plasma miR-105-5p Level

Eighty out of 319 IPD patients enrolled in this study, were of recent-onset and had not yet been treated with anti-PD medications (naive IPD), while the remaining 176 patients have been treated with various anti-PD medications, including levodopa (L-dopa) (*n* = 91) and the combination of L-dopa and dopamine receptor agonists (DR) (*n* = 85). Interestingly, both naive and anti-PD medication IPD patients showed a significant higher plasma miR-105-5p level compared to the HC (Figure 4C).

### Performance of Plasma miR-105-5p Level for IPD Diagnosis

We evaluated the performance of the expression level of miR-105-5p for the IPD diagnosis, by the AUC values, based on the ROC curve analysis. We revealed that the AUC value was 0.768 (95% CI, 0.727–0.805; *p*< 0.001) for miR-105-5p (Figure 4D) in plasma between IPD and HC.

**FIGURE 5:**
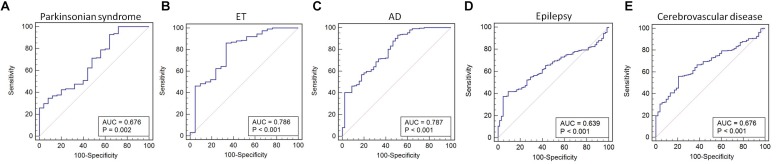
ROC curve for miR-105-5p between IPD and parkinsonian syndrome **(A)**, ET **(B)**, AD **(C)**, epilepsy **(D)**, and cerebrovascular disease **(E)**. The areas under the ROC curve (AUC) were 0.676 (95% CI, 0.615–0.732; *p* = 0.002), 0.786 (95% CI, 0.731–0.835; *p* < 0.001), 0.787 (95% CI, 0.734–0.833; *p* < 0.001), 0.639 (95% CI, 0.587–0.689; *p* < 0.001, and 0.676 (95% CI, 0.624–0.725; *p* < 0.001), respectively. ET, essential tremor; AD, Alzheimer’s disease.

We found that miR-105-5p was able to differentiate IPD from parkinsonian syndromes (AUC value: 0.676, 95% CI, 0.615–0.732; *p* = 0.002), ET (AUC value: 0.786, 95% CI, 0.731–0.835; *p* < 0.001), AD (AUC value: 0.787, 95% CI, 0.734–0.833; *p* < 0.001), epilepsy (AUC value: 0.639, 95% CI, 0.587–0.689; *p* < 0.001), and cerebrovascular disease (AUC value: 0.676, 95% CI, 0.624–0.725; *p* < 0.001) (Figure 5A–E).

## Discussion

In this study, we identified miR-105-5p as a putative biomarker for IPD using the PD specific miRNA-mRNA network and a novel bioinformatics model. We then measured the level of miR-105-5p in the plasma of a relatively larger sample size. We demonstrated for the first time that the plasma miR-105-5p level in IPD was significantly higher than that of the controls. Further analysis revealed that the AUC value of miR-105-5p in the ROC curve analysis, reached the discriminative value (AUC > 0.7) in differentiating IPD from ET and AD. These results suggest that plasma miR-105-5p could be a potential biomarker for the diagnosis of IPD.

Moreover, we observed that plasma miR-105-5p might serve as a possible biomarker to differentiate IPD from other parkinsonism. Parkinsonism is a clinical syndrome characterized by tremor, bradykinesia rigidity, and postural instability, which includes IPD and other parkinsonian syndromes (Piccini and Whone, 2004). The ability to reach a firm diagnosis and distinguish between different parkinsonism entities is of clinical importance (Hughes et al., 2001). In order to determine if the alteration of plasma miR-105-5p in IPD is specific, we specifically recruited 305 various NDC and compared the expression level of miR-105-5p in IPD with not only the HC but also the NDC. Parkinsonian syndrome (including vascular parkinsonism, multiple system atrophy, progressive supranuclear palsy, and dementia with Lewy bodies) and ET were enrolled in the NDC as the common differential diagnoses of IPD. It is especially interesting that the plasma miR-105-5p level in IPD was significantly higher than that of these diseases. ROC analyses for the diagnostic power of plasma miR-105-5p yielded an AUC of 0.786 in differentiating IPD from ET. Besides, the AUC of the plasma miR-105 was 0.676, a close value in differentiating IPD from parkinsonian syndrome. Certainly, a larger population-based study is required to further support this assumption. These results suggest that plasma miR-105-5p might be able to distinguish IPD from other diseases, especially in the differential diagnosis of IPD from ET and parkinsonian syndromes.

We further explored the effect of disease duration on miR-105-5p expression levels and found that the increased miR-105-5p expression in plasma might reflect pre-existing disease-related changes rather than a surrogate marker for disease progression. In our study, we observed that the expression levels of miR-105-5p were increased in male and female of IPD patients comparing to the same gender of control subjects. Moreover, the effects of different age groups on miR-105-5p expression were not identical. The differences between plasma miR-105-5p expression levels in HC and IPD patients were highly statistically significant in patients in the 40–59 and 60–69 age groups, but not in the 70–79 and 80–89 age groups. No statistical difference was found between the naive and anti-PD medications groups, indicating that plasma miR-105-5p might serve as a possible biomarker without the impact of anti-PD medications.

In the present study, we focused on the alteration of plasma miR-105-5p in IPD, for the potential role of circulating miRNA as a biomarker in the diseases (Teixeira dos Santos et al., 2016). Circulating cell-free miRNAs, as indicators of disease relevant information, are carried to the periphery, which can be used to monitor central nervous system diseases (Burgos et al., 2014). As a peripheral biomarker, plasma miR-105-5p may reflect pathological changes in IPD brains.

Human miR-105 is located on the intronic region of GABR^A3A^ (γ-aminobutyric acid receptor 3), which resides on the X chromosome (Benakanakere et al., 2009). The molecular function and potential target genes of miR-105 are mostly unknown. MiR-105 has been found highly expressed in human brain (Ludwig et al., 2016), suggesting that miR-105 may have a function in the central nervous system. Recent studies have shown that miR-105 could be dysregulated in human glioma tissues (Guan et al., 2015; Zhang et al., 2017). Although the relationship between miR-105 and neurodegenerative diseases has, to the best of our knowledge, not previously been reported. Growing evidence emphasizes the role of miR-105 as mediators in the inflammatory response of chronic inflammatory diseases. Fan et al. (2016) reported that the miR-105 expression level was upregulated, while lipopolysaccharide was induced in the murine macrophage cells using a miRNA qPCR array. Shen et al. (2017) showed that miR-105 modulates TNF-α-induced epithelial-mesenchymal transition in a NF-κB-dependent pathway. Recently there has been more evidence to support the role of inflammation as a measurable driving force of PD pathology (Deleidi and Gasser, 2013). We suspect that miR-105-5p may be involved in the process of PD by mediating the neuroinflammation pathway. Further studies are required to prove this assumption *in vitro* and *in vivo*.

## Author Contributions

WL conceived and supervised the study. BS conceptualized the research and developed the bioinformatics model. YC collected the data and performed the computational analysis. ZY and TL performed the experiments and collected the blood samples, drafted, and revised the manuscript. TL, ZY, SL, CC, BS, and WL revised the manuscript. All authors contributed to manuscript revision, read, and approved the submitted version.

## Conflict of Interest Statement

The authors declare that the research was conducted in the absence of any commercial or financial relationships that could be construed as a potential conflict of interest.
